# Effective connectivity associated with auditory error detection in musicians with absolute pitch

**DOI:** 10.3389/fnins.2014.00046

**Published:** 2014-03-05

**Authors:** Amy L. Parkinson, Roozbeh Behroozmand, Nadine Ibrahim, Oleg Korzyukov, Charles R. Larson, Donald A. Robin

**Affiliations:** ^1^Research Imaging Institute, Department of Neurology, University of Texas Health Science Center San AntonioSan Antonio, TX, USA; ^2^Human Brain Research Lab, Department of Neurosurgery, The University of IowaIowa City, IA, USA; ^3^Department of Communication Sciences and Disorders, Northwestern UniversityEvanston, IL, USA

**Keywords:** absolute pitch, vocalization, auditory feedback, DCM, ERP, pitch shift

## Abstract

It is advantageous to study a wide range of vocal abilities in order to fully understand how vocal control measures vary across the full spectrum. Individuals with absolute pitch (AP) are able to assign a verbal label to musical notes and have enhanced abilities in pitch identification without reliance on an external referent. In this study we used dynamic causal modeling (DCM) to model effective connectivity of ERP responses to pitch perturbation in voice auditory feedback in musicians with relative pitch (RP), AP, and non-musician controls. We identified a network compromising left and right hemisphere superior temporal gyrus (STG), primary motor cortex (M1), and premotor cortex (PM). We specified nine models and compared two main factors examining various combinations of STG involvement in feedback pitch error detection/correction process. Our results suggest that modulation of left to right STG connections are important in the identification of self-voice error and sensory motor integration in AP musicians. We also identify reduced connectivity of left hemisphere PM to STG connections in AP and RP groups during the error detection and corrections process relative to non-musicians. We suggest that this suppression may allow for enhanced connectivity relating to pitch identification in the right hemisphere in those with more precise pitch matching abilities. Musicians with enhanced pitch identification abilities likely have an improved auditory error detection and correction system involving connectivity of STG regions. Our findings here also suggest that individuals with AP are more adept at using feedback related to pitch from the right hemisphere.

## Introduction

Understanding the neural mechanisms underlying human vocalization provides insight into sensory motor control that can inform voice production in health and disease. A critical need is the development of neurbiologically plausible models of vocalization that apply to a wide range of perceptual abilities (disordered-average-professional) in order to fully understand how vocal control varies across the full spectrum of abilities. From a system level perspective, understanding the regions involved in vocalization cannot provide information about the neural networks that govern the wide range of sensory motor interactions that lead to vocal output. Rather, it is necessary to study how regions of the brain are functionally connected within the voice production system. Prior studies (Bengtsson et al., 2005; Han et al., 2009; Loui and Schlaug, 2009; Kleber et al., 2010; Halwani et al., 2011) have shown differences between musicians and non-musicians while performing motor, auditory or somatosensory tasks. It is also known that voluntary responses to shifts in vocal pitch are more accurate and stable in experienced singers compared to non-musicians (Zarate and Zatorre, 2008), suggesting enhanced sensory control over the voice. A rare but interesting ability is perfect or absolute pitch (AP), which is the ability to perceive and identify exact musical notes. Individuals with AP have an enhanced ability to accurately relate a note to a musical scale without an acoustical reference pitch (Takeuchi and Hulse, 1993). The behavioral characteristics of AP have been examined extensively although the etiology is still unknown. Importantly, differences in both structural and functional characteristics of the brains of individuals with AP when compared to controls who do not possess AP have also been identified (Schlaug et al., 1995; Schlaug, 2001; Bermudez et al., 2009; Loui et al., 2011; Dohn et al., 2013). Specifically structural imaging studies have identified a stronger leftward asymmetry of the planum temporale when comparing AP musicians with non-AP musicians (Schlaug et al., 1995). Functional imaging studies have also identified differences in AP, specifically inferior frontal (Zatorre et al., 1998) and superior temporal regions as being increasingly activated in AP musicians during tone perception and pitch memory tasks (Schulze et al., 2009, 2012).

Given the unique ability of AP, we expect that such enhancement of the functional mechanisms underlying sensory control of the voice in trained singers may lead to adaptations in the functional connectivity of brain regions and networks specifically related to audio-vocal integration and voice control. To date, there is one study that has examined functional connectivity networks in people with AP (Loui et al., 2012). Loui et al. (2012) used graph theory analysis of fMRI data to examine networks of functional activation during music listening. Results identified increased clustering in the left superior temporal regions in AP subjects compared to controls. However, to provide data on sensory control mechanisms of vocalization across the spectrum of abilities we have used a pitch perturbation approach in which a pitch-shift is introduced during vocalization (Larson, 1998). This approach has provided exceptionally robust data allowing for detailed insight into human vocalization.

In the present experiment we studied effective connectivity in musicians with AP compared to musicians with relative pitch (RP) and subjects with no musical ability (NM) using dynamic causal modeling (DCM) to model effective connectivity of ERP responses to pitch shifted auditory feedback. We have previously identified bilateral STG regions as playing a key role in sensory control of the voice. Using both fMRI and DCM methods we have identified the importance of STG in sensory motor control during pitch-shifted stimuli in healthy young subjects (Parkinson et al., 2012, 2013). Here we compared families of models examining STG involvement in the error detection/correction process. Based on the work discussed above, our *a priori* hypothesis was that DCM modeling would identify different connectivity patterns in individuals with AP when compared to RP and NM individuals. Based on previous literature of structural and functional differences in the AP brain (Schlaug et al., 1995; Bengtsson et al., 2005; Han et al., 2009; Loui and Schlaug, 2009; Kleber et al., 2010; Halwani et al., 2011; Loui et al., 2012). We expected to see differences specifically in the left hemisphere connections between STG and IFG/PM, during vocalization with pitch-shifted feedback. We also expected to see a difference in the modulation of connections between the left and right hemisphere STG regions based on our previous work (Zarate and Zatorre, 2008; Parkinson et al., 2012, 2013; Behroozmand et al., 2014). This work will provide additional understanding of the brain networks related to a range of sensory motor perceptual skills and an insight into the neural mechanisms driving differences in pitch perception across a spectrum of ability.

## Materials and methods

### Participants

Thirty-three speakers of American English (18 females and 15 males, ages 18–25 years) with no history of neurological disorder participated in the study. Absolute and relative pitch subjects were recruited from the Bienen School of Music and the untrained non-musicians were recruited from the general Northwestern University student population. There were 11 subjects recruited to each of the AP, RP, and untrained non-musician (NM) groups. All musicians had a minimum of 4 years musical training [*AP* = 12.23 years (mean), range 7–16 years, *RP* = 11.64 years (mean), range 4–17 years]. Within the musician groups the instruments played included guitar, piano, violin, cello, clarinet, saxophone, trombone, trumpet, tuba, bassoon, French horn, oboe, and flute.

A bilateral pure-tone hearing-screening test at 20 dB SPL (octave frequencies between 250 and 8000 Hz) was conducted to screen for normal hearing. A test of musical proficiency was conducted on the participants to evaluate their degree of pitch perception, identification, discrimination, and production abilities. The test included evaluation of chromatic pitch identification, chromatic sight singing, atonal sight singing, and microtonal pitch identification (for detailed description of the tests please see Behroozmand et al., 2014). Each subject's performance across all tests was evaluated and each subject was given an objective rating score between 0 and 100%. Classification into groups was based on score, with individuals classified as NM scoring below 50%, individuals classified as RP musicians scoring between 50 and 90% and individuals classified as AP scoring over 90%.

The Northwestern University institutional review board approved all study procedures including recruitment, data acquisition and informed consent, and subjects were monetarily compensated for their participation. Written informed consent was received from all participants.

### Experimental design

**Figure 1:**
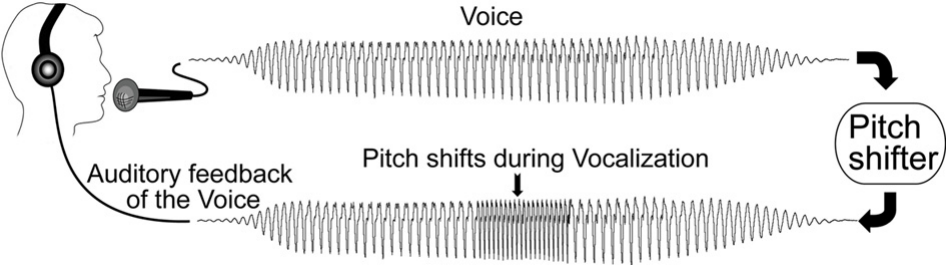
**Schematic illustration of the voice perturbation paradigm adapted from Korzyukov et al. (2012)**.

During the experimental session, subjects were seated in a sound-treated room and were instructed to sustain the vowel sound /a/ for approximately 2 s. Subjects were asked to vocalize at their conversational pitch and loudness levels whenever they felt comfortable, i.e., without a cue. Subjects were informed that their voice would be played back to them through headphones during their vocalizations. Subjects were instructed to ignore any pitch-shifts they heard in the feedback of their voice. There was a pause of around 1–2 s between vocalizations, which allowed subjects to take a breath. During each vocalization a pitch-shift stimulus (±100 cents, 200 ms duration) was presented (Figure 1) in the auditory feedback occurring between 500 and 1000 ms after voice onset. All pitch-shift stimuli were randomly varied between type and pitch-shift onset from trial to trial. The unit, “cents” is a logarithmic value related to the 12-tone musical scale, where 100 cents equals one semitone. The rise time of the pitch shift was 10–15 ms. In each block of trials there were 120 vocalizations taking approximately 15–20 min. The experiment consisted of two blocks of trials for a total experiment duration of approximately 30–40 min. Subjects were asked to keep their eyes open throughout the recording session.

Subjects' voices were picked up with an AKG boomset microphone (model C420), and amplified with a Mackie mixer (model 1202-VLZ3). Pitch shifting of the voice was performed using an Eventide Eclipse Harmonizer. MIDI software (Max/MSP v.5.0 Cycling 74) was used to control the time delay of the shift from vocal onset, the duration, direction, and magnitude of pitch shifts. Voice and auditory feedback were sampled at 10 kHz and recorded onto a laboratory computer utilizing Chart software (AD Instruments) and a PowerLab A/D Converter (Model ML880, AD Instruments). Subjects maintained their conversational F0 levels and voice loudness (about 70–75 dB) throughout the experiment, and the feedback signal (i.e., the subject's pitch-shifted voice) was delivered back to the subjects through Etymotic earphones (model ER1-14A) at a loudness of about 80–85 dB. The 10 dB increase in loudness between voice and feedback channels (controlled by a Crown amplifier D75) was used to partially mask air-born and bone-conducted voice feedback.

### EEG acquisition

The electroencephalogram (EEG) signals were recorded from 32 sites on the subject's scalp using an Ag-AgCl electrode cap (EasyCap GmbH, Germany) in accordance with the extended international 10–20 system (Takeuchi and Hulse, 1993; Oostenveld and Praamstra, 2001) including left and right mastoids. Electrode impedances were kept below 5 kΩ for all channels. EEG recordings were made using the average reference montage in which outputs of all of the amplifier channels were averaged. This averaged signal was used as the common reference for each channel. Signals were low-pass filtered with a 400-Hz cut-off frequency (anti-aliasing filter), digitized at 2 kHz, and recorded using a BrainVision QuickAmp amplifier (Brain Products GmbH, Germany). Electro-oculogram (EOG) signals were recorded using two pairs of bipolar electrodes placed above and below the right eye to monitor vertical eye movements and at the canthus of each eye to monitor horizontal eye movements.

### Data analysis

#### ERP analysis

SPM8 [http://www.fil.ion.ucl.ac.uk/spm; update number 4667, (Schlaug et al., 1995; Schlaug, 2001; Bermudez et al., 2009; Litvak et al., 2011; Loui et al., 2011; Dohn et al., 2013)] was used for all ERP pre-processing and data analysis. The data were first epoched into to single trials, with a peri-stimulus window of −100 to 500 ms. The data were then down-sampled to 128 Hz and band-pass filtered (Butterworth) between 0.5 and 30 Hz. Artifact removal was implemented with robust averaging. A minimum number of 100 epochs were averaged for each condition. The data were finally grand averaged over 11 AP musicians, 11 RP musicians, and 11 non-musician subjects.

#### DCM analysis

SPM8 was also used to perform DCM (Schlaug et al., 1995; David et al., 2006) on the data. DCM was used to examine the connections between neural regions involved in the proposed model of processing auditory feedback during vocalization. DCM in SPM was originally created to analyze effective connectivity of fMRI data, and subsequently this was extended to model ERPs (Zatorre et al., 1998; David et al., 2006; Kiebel et al., 2006). The DCM method uses neural mass models to describe neural activity and estimate effective connectivity within a specified network model. Source time courses are first generated by a neurobiologically realistic model of the network of interest. These are then projected on the scalp using a spatial forward model (Boundary Element Model in our case). The parameters of both the source model and the neural model are optimized using a variational Bayesian approach to match the observed EEG data as closely as possible. Data were modeled within a time-window of 1–200 ms following the pitch-shift stimulus with an onset of 60 ms. A Hanning window was applied to the data and a detrend parameter of 1 was used with 8 modes. The evoked responses were modeled using the IMG (imaging) option, which models each source as a patch on the cortical surface (Daunizeau et al., 2009; Schulze et al., 2009, 2012). The data for each of the three subject groups were modeled separately. For each pitch-shift direction (up and down) conditions were modeled together allowing particular connections in the model to vary to explain the difference between the two.

#### Model identification and selection

**Figure 2:**
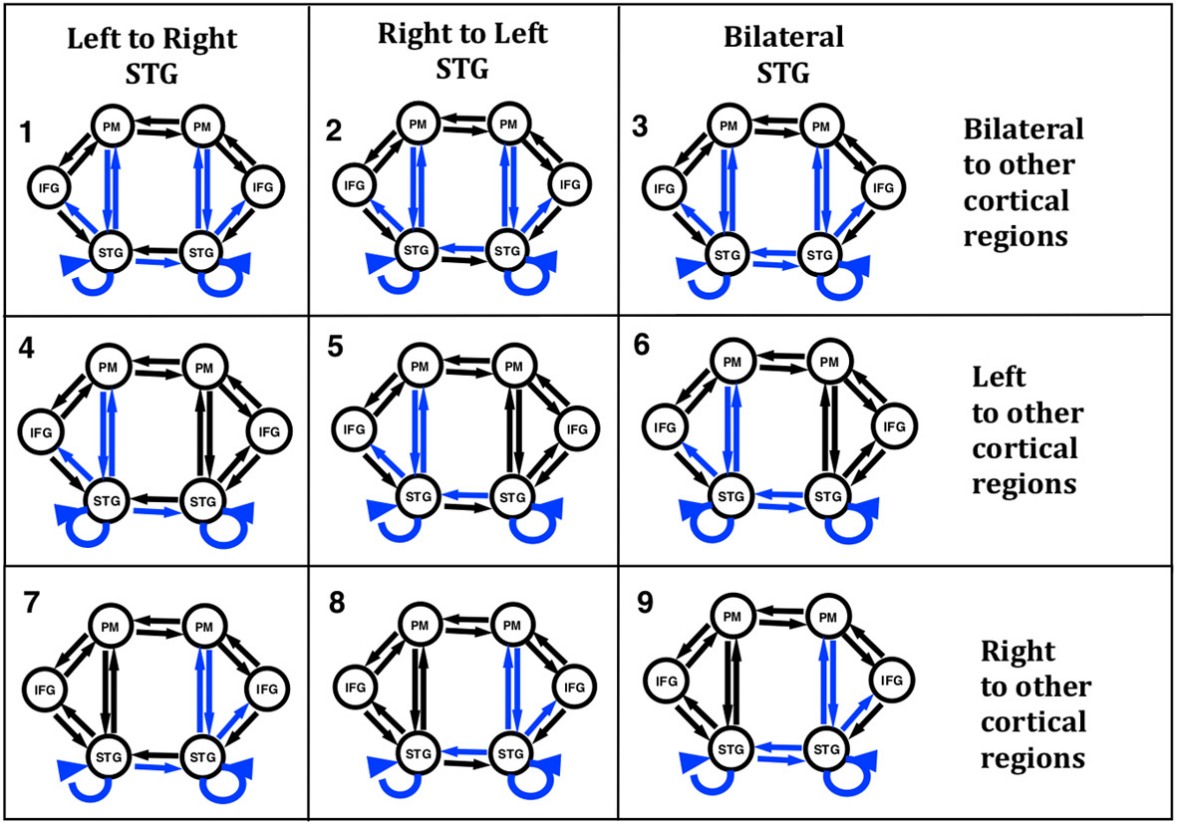
**Nine versions of the model were combined into families and analyzed for all three subject groups**. The input to the model was set to the STG regions. Model families varied based on one of two factors; (i) differences in cross hemisphere STG connections being modulated or (ii) bilateral, left, or right connections between STG, PM, and IFG being modulated. The basic model is represented by black arrows with blue arrows identifying modulated connections.

In order to test our hypotheses with DCM we constructed models with six regions and 18 connections. While there are many different models that could have been examined, we chose our model structure based on the literature and our initial work to address questions regarding the role of the STG in the identification of self voice error. Our model regions and network architecture for this experiment was motivated by results from a previous fMRI and ERP-DCM studies of pitch-shifted vocalization (Loui et al., 2012; Parkinson et al., 2012, 2013). The peak MNI coordinates reported in the literature for vocalization and those modeled in our previous ERP-DCM study (Larson, 1998; Parkinson et al., 2012, 2013) were used as coordinates for source regions for the models examined here. Three regions were selected in both the left and right hemispheres. The regions were superior temporal gyrus (STG), inferior frontal gyrus (IFG), and premotor (PM) cortex. MNI coordinates of the regions are displayed in Table 1. The basic model selected for analysis included modulated connections from STG to PM, PM to STG, and STG to IFG in both hemispheres. Variations in modulations across hemisphere from STG to STG and from STG to other cortical regions (PM and IFG) were examined. We specified a bilateral driving input to STG as the starting point to the model and nine different variations of the model (Figure 2) were examined.

**Table 1:** **Source location coordinates in MNI space**.

**Sources**	**Coordinate (*x*, *y*, *z*)**
Left STG	−59, −16, 6
Right STG	63, −11, 6
Left PM	−57, 2, 30
Right PM	60, 14, 34
Left IFG	−32, 31, 3
Right IFG	56, 32, 24

In the present study we proposed to examine differences in connectivity across hemispheres between the left and right STG regions. Reasoning behind examining lateral STG connectivity was based on our previous study using DCM to model vocal responses to pitch shifted voice feedback (Parkinson et al., 2012, 2013) and evidence of both structural and functional differences in STG regions in AP musicians (Schulze et al., 2009; Loui et al., 2011, 2012). Lateral STG connectivity was the first model characteristic we chose to examine (factor 1). For this analysis we split the nine models into three different families consisting of either left to right STG (LtoR), right to left STG (RtoL) or bilateral (Bilat) connections being modulated by the experimental effect (shifted vocalization, upwards vs. downwards shifts). All other model parameters were identical (Table 2, Figure 2).

**Table 2:** **Two separate analyses of model families were performed**.

**Analysis**	**Family name and description**	**Models included in family**
Factor 1 Effect of STG modulation across hemispheres	LtoR—Models with left to right STG modulated	1, 4, 7
	RtoL—Models with right to left STG modulated	2, 5, 8
	Both—Models with left to right and right to Left STG modulated	3, 6, 9
Factor 2 Effect of bilateral, left or right connections	Bilat—Bilateral connections between STG, PM, and IFG modulated	1, 2, 3
	Left—Only Left hemisphere connections between STG, PM, and IFG modulated	4, 5, 6
	Right—Only Right connections between STG, PM, and IFG modulated	7, 8, 9

The second characteristic we chose to model (factor 2) was the effect of connectivity between regions within a hemisphere. Reasoning behind this was again based on previous literature identifying differences in the left hemisphere superior temporal regions both functionally and structurally (Schulze et al., 2009; Loui et al., 2011, 2012), in individuals with AP. The right hemisphere is also known to be involved in pitch processing (Divenyi and Robinson, 1989; Binder et al., 1997; Johnsrude et al., 2000; Zatorre and Belin, 2001). Based on this literature we specified connections between STG to PM, PM to STG, and STG to IFG as being modulated by the experimental effect. We examined three different families of models where we specified only left hemisphere, only right hemisphere or both left and right hemispheres (bilateral) connections as being modulated by the experimental effect (Table 2, Figure 2).

Model comparison was performed for each of the three groups separately with a Bayesian model selection (BMS) family level inference procedure (Penny et al., 2010). Family level inference identifies the “best family of models” which is the one with the highest log-evidence for a given family over the other families across subjects. We used BMS for random effects (Stephan et al., 2009) to compare families across each of our two factors examined for each group (Table 2, Figure 2). Family model inference removes uncertainty about certain aspects of model structure other than the specific factor of interest. Family model inference outputs a model exceedance probability for each family of models examined. The family of models with the highest exceedance probability, i.e., the highest relative probability compared to any other model tested, was identified as the family which best represented the data. We then used these identified models to make inference about model structure across the groups.

## Results

### Behavioral and ERP results

**Figure 3:**
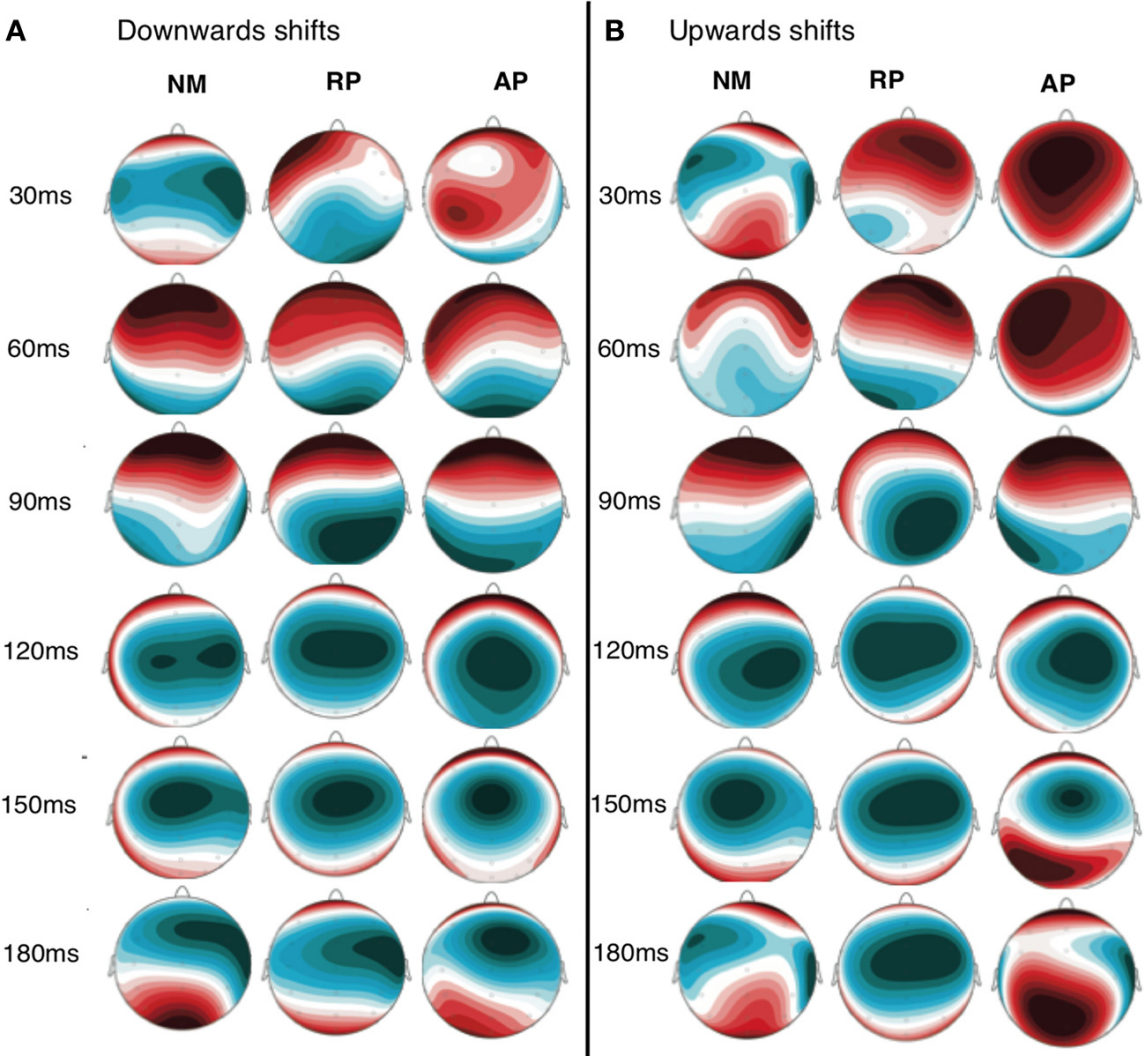
**Scalp distribution map of grand average activity for both downwards (A) and upwards (B) shift conditions for all three groups**. Time points between 30 and 180 ms are displayed to identify the distribution of scalp potentials during the time period selected for DCM analysis.

**Figure 4:**
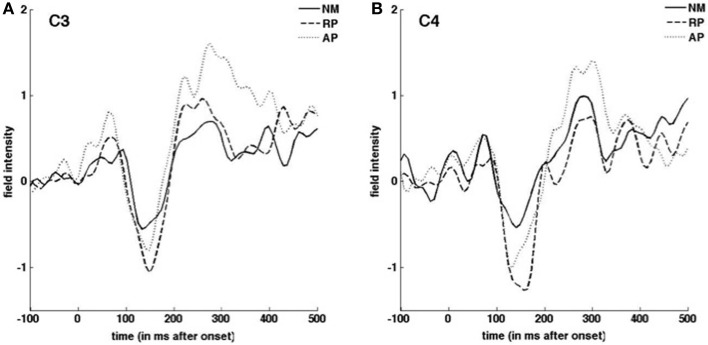
**Grand average ERP responses to downward pitch-shifted voice feedback of 100 cents**. Responses from C3 **(A)** C4 **(B)** EEG channels are shown for NM (solid line), RP (dashed line), and AP (dotted line) groups.

ERP and vocal responses to pitch shifted stimuli are well established in the literature (Behroozmand and Larson, 2011; Liu et al., 2011; Korzyukov et al., 2012). Further detailed analysis of vocal and ERP responses from this data set are already published (Behroozmand et al., 2014). Figure 3 identifies scalp potential distribution of responses for all three groups to the up and down stimuli for the 1–200 ms post stimulus onset timeframe. The spatial variation seen in this figure provides justification for including the separate nodes in the DCM analysis. Grand average responses for all three groups for the 100 cent shift down condition is shown in Figure 4 where differences in both N1 and P2 responses can be seen across the groups. Responses from the left hemisphere C3 (Figure 4A) and right hemisphere C4 (Figure 4B) channels are displayed. The variation in responses between groups and across hemispheres again provides justification for examining left and right hemispheres separately across the three groups.

### DCM results

#### Factor 1—effect of STG modulation across hemispheres

**Figure 5:**
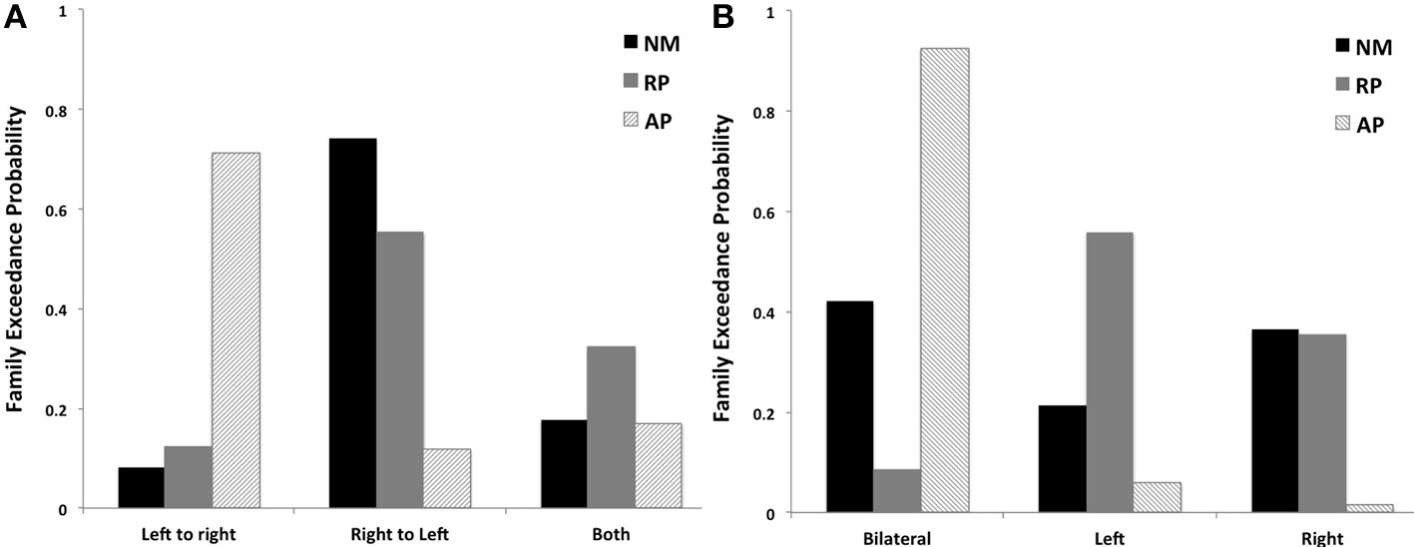
**BMS results for families of models examining (A) effect of STG connections across hemispheres and (B) effects of laterality of STG connections to other cortical regions for 3 subjects groups**.

Although there were no significant winning families identified for each group, BMS of the three families examining factor 1 identified that the AP group favored models with left to right STG connections (LtoR, models 1, 4, and 7, as displayed in Figure 2) being modulated (0.71 LtoR, 0.12 RtoL and 0.17 both random effects model exceedance probability). The RP and NM groups both favored the family with right to left STG connections (RtoL, models 2, 5, and 8, as displayed in Figure 2) modulated (Figure 5A) (RP group: 0.12 LtoR, 0.55 RtoL and 0.32 both random effects model exceedance probability; NM group: 0.08 LtoR, 0.74 RtoL and 0.18 both random effects model exceedance probability).

#### Factor 2—effect of bilateral, left, or right connections

The AP group clearly favored models with bilateral connections to other cortical regions being modulated (bilateral, models 1, 2, and 3, as displayed in Figure 2) (0.93 bilateral, 0.06 left and 0.01 right random effects model exceedance probability). In comparison the RP and NM groups did not significantly favor one family of models over another (Figure 5B) (RP group: 0.09 bilateral, 0.56 left and 0.35 right random effects model exceedance probability; NM group: 0.42 bilateral, 0.21 left and 0.37 right random effects model exceedance probability).

#### Influence on coupling

**Figure 6:**
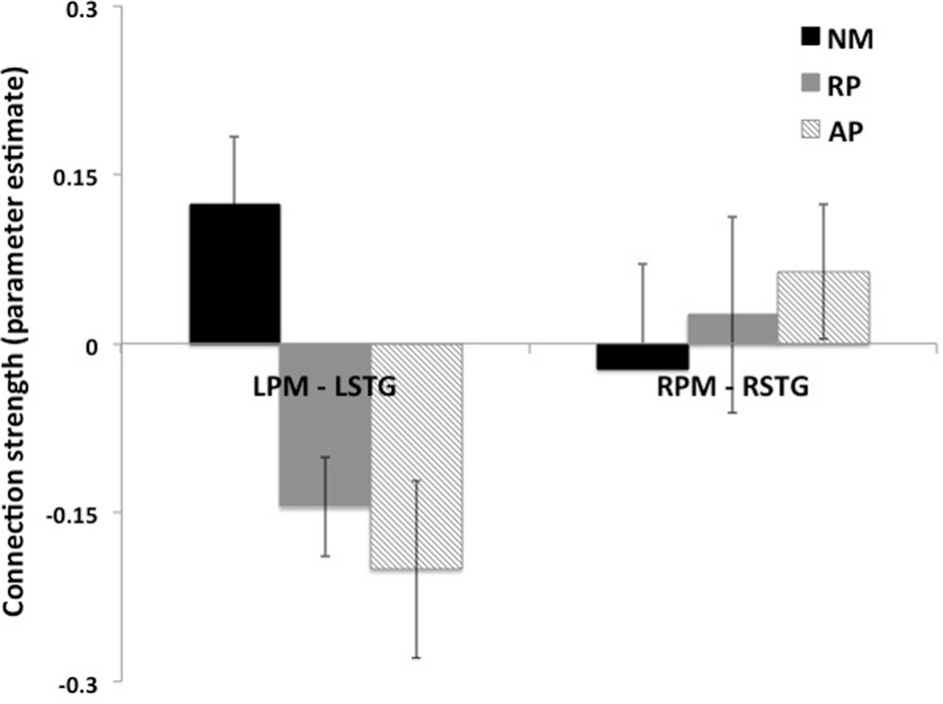
**Difference in connection strengths for PM to STG connections for all three groups**.

Significance of coupling parameters were directly compared across all groups for all modulated connections of the bilateral family of models. Bayesian model averaging (BMA) was performed to identify coupling parameters for all connections within this family of models for every subject. Analysis of the coupling parameters derived from BMA showed a group specific modulation of the connections between left PM and left STG nodes (Figure 6), with a negative coupling between these nodes seen in the AP and RP groups and a positive coupling in the NM group (*p* < 0.05, 2 sample *t*-test). No difference was seen in coupling strength of the right hemisphere PM to STG connection.

## Discussion

The present study examined the effective connectivity of the neural networks associated with processing voice auditory feedback in individuals with varying pitch processing and identification abilities. Musicians have enhanced pitch identification mechanisms used for evaluating both vocal or instrument output resulting from continued practice. This enhanced pitch processing ability could be the result of stronger coupling between auditory-vocal motor networks for enhanced integration of feedback to update the predictive or feedforward internal model. The development of an internal representation of pitch in AP musicians may also be associated with their improved feedback-based monitoring and control of voice through more precise predictions of self-produced pitch provided by the efference copies of the motor commands. Online integration of auditory feedback to update the forward model must be essential for any musician and has likely been further enhanced and improved over years of practice and evaluation of performance. We have previously identified the STG as playing a key role in voice error detection and correction (Parkinson et al., 2012, 2013) and STG has also been identified as a critical region in AP (Loui et al., 2012; Schulze et al., 2012; Dohn et al., 2013). Here we asked questions relating to lateral STG connectivity and connectivity of STG to PM and IFG connections in each hemisphere during pitch shifted auditory feedback. Our findings indicated that modulation of STG connections to PM and IFG in both hemispheres is critical in the identification of self-voice pitch error in musicians with AP but not in the RP and NM groups. We also identified reduced connectivity of left hemisphere PM to STG connections in AP and RP groups, compared to a positive coupling in the NM group during the error detection and corrections process. When examining lateral STG connectivity we showed that individuals with AP favor models with modulations in left to right connectivity whereas both RP and NM groups favored models with modulation in right to left STG connectivity. Finally, we note that the cohort of musicians in this study included those who played instruments and expert voice users, thereby suggesting that the pitch-shift vocalization paradigm has applications relative to the study of auditory feedback across a variety of voice and non-voice areas.

The main finding of the current study identified the importance of left hemisphere connections from PM to STG in musicians during auditory error detection and correction. A considerable amount of evidence identifies the role of the left hemisphere and superior temporal regions in AP. Specifically, hemispheric differences in both brain structure and function have been identified in individuals with AP compared to controls (Bermudez and Zatorre, 2009; Loui et al., 2011; Schulze et al., 2012). Diffusion tensor imaging (DTI) studies have shown increases in white matter connectivity between the STG and middle temporal gyrus especially in the left hemisphere in AP individuals (Loui et al., 2011). The planum temporale has also been identified in AP as a region showing increased volume in individuals with AP (Zatorre et al., 1998) and altered left-right asymmetry when comparing AP musicians with non-AP controls (Schlaug et al., 1995; Zatorre et al., 1998; Keenan et al., 2001; Dohn et al., 2013). Studies examining functional brain activations in AP musicians have also identified left hemisphere differences in AP, specifically inferior frontal (Zatorre et al., 1998) and superior temporal regions as increasingly activated during tone perception and pitch memory tasks (Schulze et al., 2009, 2012). It is likely that a predictive model of vocal output is created in the left hemisphere. Auditory feedback related to spectral (pitch) and temporal components of the voice is then compared with the predicted model. The motor output and forward model are then corrected and updated should any error signals arise between predicted and actual feedback. It is likely that musicians with enhanced abilities to accurately relate a note to a musical scale likely have an improved error detection and correction system. This would result in more precise internal models through years of practice and “fine-tuning” of the system and therefore these individuals rely less upon integration of feedback from premotor regions in the left hemisphere to update and maintain a current representation in this model.

One key observation relates to the nature of the difference in modulation of left hemisphere PM to STG connection between the groups. Both musician groups (AP and RP) showed a negative coupling between these regions compared to non-musician controls who showed a positive coupling, suggesting that this connection is inhibitory in both musician groups (Figure 6). Thus, the role of the left hemisphere in error detection/correction mechanisms may be functionally different in musicians than in non-musicians. The inhibitory connection seen here between left PM and STG regions in musicians, suggests that STG activity is regulated by a frontal control system that assists in fine-tuning sensory motor integration. We have previously shown that left to right STG connections are key in pitch error detection and correction (Parkinson et al., 2013) and here that this connection is carefully tuned by inhibition from PM. Furthermore we also found evidence of bilateral connectivity of STG to both PM and IFG in AP only, suggesting a need for greater interhemispheric interplay in this subject group.

The right hemisphere auditory areas have long been shown to be responsible for the processing of pitch. Examination of the specialization of the auditory cortex and STG to both spectral and temporal information has shown that damage to the right hemisphere STG affects a variety of pitch related processing tasks (Zatorre, 1985; Divenyi and Robinson, 1989; Robin et al., 1990). Specifically lesions to the right but not left primary auditory cortical areas impaired processing of pitch change (Johnsrude et al., 2000). The role of the right hemisphere in voice control in individuals with enhanced pitch processing abilities is unclear but it is likely linked to exquisite pitch discrimination and providing feedback to update and correct predictive models. Improved pitch error detection in the AP brain could reflect the development of stronger neural representations of pitch, facilitated by efference copies of the vocal motor system. Our findings here may suggest that individuals with AP are more adept at integrating feedback related to pitch from the right hemisphere.

While it is clear that both the left and right hemispheres are involved in vocal pitch error detection and correction processes as identified here, different processing demands between individuals with varying pitch matching ability result in causal network coupling differences across groups. Another observation from our results relates to differences in lateral STG connectivity between groups. While not significant, it is clear that the groups favored different models. The AP group favors models with left to right lateral STG coupling compared to RP and NM groups who favored models with right to left STG coupling. This provides further evidence that individuals with AP have enhanced pitch memory and representation of the fundamental features of the pitch leading to a more accurate prediction, which facilitates their use of the left hemisphere more in the corrective process. Because the AP brain is so highly analytic there is less need for integration of information from the right hemisphere to update predictive models in the left hemisphere. Thus, the integration of feedback into the forward model might be through lateral STG connectivity, updating information based on pitch feedback (from the right hemisphere) and temporal components (from the left hemisphere) and with fine-tuning from an inhibitory left PM to STG connection.

The existing literature on network connectivity in AP has been performed using graph theory analysis to examine functional and structural network properties (Jäncke et al., 2012; Loui et al., 2012). Loui et al. (2012) identified increased functional activation, network clustering and efficiency of connections in the left STG region in AP. The present study is the first to use DCM of event related potentials (ERP's) in musicians to take advantage of the exceptional temporal resolution of electrophysiological signals for more precise modeling of temporal dynamics within a network of specified brain regions. Our findings support the notion of experienced musicians being highly skilled at monitoring auditory feedback in order to regulate vocal or instrument output during performance. Individuals with AP have an enhanced pitch mismatch detection system, which is sensitive to the very smallest changes in pitch. It could also be the case that individuals with AP are able to retain information relating to the pitch of a note in their long-term memory and therefore have a more accurate internal representation of the pitch used in the comparison of actual and predicted auditory feedback when identifying an unknown pitch. On the opposite end of the spectrum to individuals with enhanced pitch processing skills is the disorder of congenital amusia where individuals affected are unable to detect out-of-key tones and are aware when others (or themselves) sing out of tune. Behavioral investigation of the disorder has linked the impairment to a deficit in pitch processing (Foxton et al., 2004; Hyde and Peretz, 2004). DCM of IFG and auditory cortex during melody encoding revealed increased lateral auditory cortex connectivity and a reduction in coupling in the right hemisphere IFG to auditory cortex in aumsics relative to control subjects (Albouy et al., 2013). This result of a reduction in coupling the right hemisphere in individuals at the opposite end of the pitch perception skill spectrum provides further support for our hypothesis of increased involvement of the right hemisphere for pitch detection in AP yet reduced need for lateral connectivity to integrate information due to a more precise initial model.

Finally, we observe limitations in the current study. We recognize that more optimal network models involving additional brain regions (e.g., supplementary and primary motor regions) may exist in regard to vocal error detection and correction mechanisms in musicians. We based the current models on *a priori* hypotheses and only tested connections specific to these. It may be the case that experienced musicians recruit additional or alternative brain regions that we did not test. Due to the limitation in number of regions included in a DCM it is not possible to perform a direct comparison of many regions across both hemispheres. Also, the analysis we performed examined a time-window of 1–200 ms-post onset of the pitch shift. This time window chosen for analysis may not be the optimal timeframe to reflect pitch processing. An additional analysis with an extended time window (1–400 ms) could be performed to examine the effect of later components.

In using DCM of ERP data we have shown reduced connectivity between left PM and STG regions in individuals with enhanced pitch processing abilities compared to non-musician controls. We also identified differing lateral STG connectivity and hemispheric involvement related to pitch matching ability. These results provide further support for the involvement of STG in vocal pitch error detection and correction and also provide insight into the network and hemisphere differences in individuals with highly enhanced error discrimination abilities. That our subjects were not necessarily singers strongly suggests that the pitch-shift vocalization paradigm can be used to understand auditory motor integration in general rather than for vocalization or speech only.

## Author contributions

The original ERP research project was designed by Charles R. Larson, Roozbeh Behroozmand, Nadine Ibrahim, and Oleg Korzyukov. The DCM modeling portion of the project was designed by Amy L. Parkinson and Donald A. Robin. Research project organization and data collection was performed by Nadine Ibrahim, Roozbeh Behroozmand, and Oleg Korzyukov. Data analysis was performed by Amy L. Parkinson. The first draft of the manuscript was prepared by Amy L. Parkinson and all authors were involved in manuscript review, critique, and revision.

### Conflict of interest statement

The authors declare that the research was conducted in the absence of any commercial or financial relationships that could be construed as a potential conflict of interest.
